# Distortion Calculation Method Based on Image Processing for Automobile Lateral Mirrors

**DOI:** 10.3390/mi13030401

**Published:** 2022-02-28

**Authors:** Carlos Paredes-Orta, Luis M. Valentin-Coronado, Arturo Díaz-Ponce, Juvenal Rodríguez-Reséndiz, Jorge Domingo Mendiola-Santibañez

**Affiliations:** 1 CONACYT—Centro de Investigaciones en Optica, Unidad Aguascalientes, Prol. Constitución 607, Reserva Loma Bonita, Aguascalientes 20200, Mexico; cparedes@cio.mx (C.P.-O.); luismvc@cio.mx (L.M.V.-C.); adiaz@cio.mx (A.D.-P.); 2 Facultad de Ingeniería, Universidad Autónoma de Querétaro, Santiago de Querétaro 76010, Mexico; juvenal@uaq.mx

**Keywords:** mixed techniques, mechatronics, convex mirrors, circularity, distortion factor, shape analysis

## Abstract

The automobile lateral-view mirrors are the most important visual support for driver safety; therefore, it is important they have robust quality control. Typically, the distortion of a lateral-view mirror is measured using the JIS-D-5705 standard; however, this methodology requires an expert person to perform the measurements and calculations manually, which can induce measurement errors. In this work, a semi-automatic distortion calculation method based on image processing is presented. Distortion calculations of five commercial mirrors from different manufacturers were performed, and a comparative study was carried out between the JIS-D-5705 standard and the proposed method. Experimental results performed according to the JIS-D-5705 standard showed that all mirrors have a distortion lower than 5%, indicating that all meet the standard. On the other hand, the proposed method was able to detect that one of the mirrors presented an important distortion, which was not detected by the methodology proposed in the standard; therefore, that mirror should not meet the standard. Then, it was possible to conclude that the proposed distortion calculation method, based on image processing, has higher robustness and precision than the standard. In addition, an appropriate and effective behavior against changes in scale, resolution, and, unlike the standard, against changes in image rotation was also shown.

## 1. Introduction

In the automotive industry, one of the most important parts in the vehicle, in terms of visual driving aid, are the lateral-view mirrors. Lateral-view mirrors play a very important role when driving, as they provide the functionality of displaying the left and right sides of the vehicle [1]. These mirrors help us to reflect the edge of the car body, and at the same time, we can see part of the environment around us. Commonly, these mirrors are convex and give a wide field of view, projecting an image of the vehicles that circulate. Nevertheless, the projected image of the objects can be seen smaller than they really are, and that is why it is common to observe the legend “Objects in mirror are closer than they appear”. Then, it is very important that manufacturers guarantee a good quality of mirrors so that the safety, as well as the driving experience, may be improved [1].

**Figure 1:**
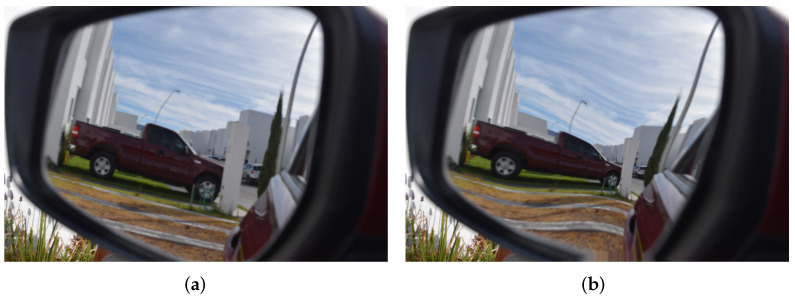
Example of an image of a lateral-view mirror. (**a**) Normal mirror image. (**b**) Mirror image with distortion.

The final process of lateral-view mirror manufacturing is quality inspection. The quality of a mirror is defined by three parameters: radius of curvature, reflection, and distortion effect [2,3]. To measure the mirror quality, an expert person performs a manual inspection, commonly by one of these two methods: concentric circle patterns or radial line patterns [4,5]. For these two methods, an image with a concentric or radial pattern is captured by a digital camera; then, this captured pattern is compared against the original one to calculate a distortion factor. There is a common standard for the measurement of this distortion factor, the JIS-D-5705 standard [4]. However, implementing these standards can be complex, time-consuming, and error-prone because, typically, the necessary measurements are done manually and even if the person is an expert, the measurements are slow and have low precision. Furthermore, as will be discussed in the next section, this standard is not rotation invariant, since it only measure eight points to generate a quality criterion, so it is possible that some areas with considerable distortions may not be taken into account. Figure 1 shows both a typical lateral-view mirror image and an image with common mirror distortion; as can be seen, the distortion changed the size of the truck.

An efficient alternative to automatically calculate the distortion factor can be implemented through digital image processing algorithms [6,7], particularly using mathematical morphology, since this method is fundamentally based on the analysis of the shape. In order to do that, a shape measure is needed, for example, circularity. Then, the distortion factor can be related to the distortion that a circle undergoes once it has been reflected in the mirror.

There are different ways to calculate circularity; its essential properties can be found in [8,9], among the most used is the classical measure of shape factor, which relates the area and the perimeter of the shape. However, this measure is not scale-invariant, is very sensitive to small irregularities, and is sensitive to the resolution [10]. To address these drawbacks, some other methods have been proposed, such as radius ratio (RR) [11], which calculates the ratio between the minimum and maximum radius of the object, or the mean circularity (MR) [11,12], where the average radius from the border points is needed. Even though these two methods improve the previous one, their use is intended for cases with regular objects, and in cases where there is partial information about the object or abrupt variations in the shape, these measurements are not very reliable. As an alternative for the measurement of circularity, the authors in [8,13] have proposed a strategy based on a non-parametric stochastic approach, that is, instead of directly considering the geometry of the shape (area and perimeter), they proposed the use of a distance distribution between the border points and the center of the shape. Then, they compare this distribution with the one given by an “ideal” circle that they infer.

Considering the mentioned disadvantages of the manual inspection of automobile lateral-view mirrors and promoting automation under a reliable, robust and precise method, this work proposes a distortion calculation method based on image processing (DCMIP), which is both scale and rotation invariant, as well as being robust to changes in the image resolution. The remaining of the paper is organized as follows. In Section 2, the calculation of the distortion factor based on the JIS-D-5705 standard is presented. Section 3 and Section 4 detail the proposed mirror DCMIP and its evaluation, respectively. Finally, the conclusions as well as the future work is presented in Section 5.

## 2. Mirror Distortion Calculation Based on the JIS-D-5705 Standard

**Figure 2:**
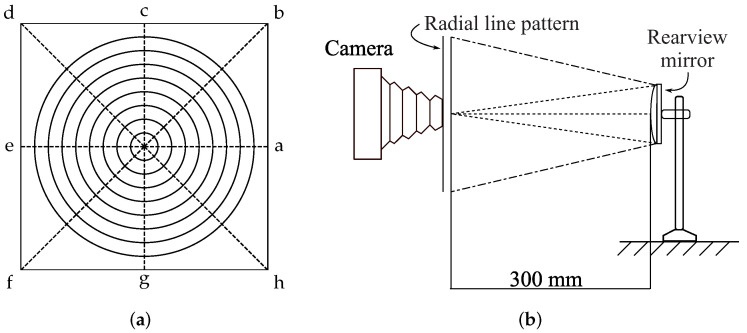
Setup to perform the distortion measurement according to JIS-554321 standard: (**a**) radial line pattern and (**b**) lateral-view setup.

Distortion of a lateral-view mirror image can be defined as a change, twist, or lack of proportionality. One of the most common vehicle mirror deformations is due to their convexity, which was intentionally manufactured in this way to widen the driver’s field of view; however, the driver loses distance perception accuracy. If the degree of distortion is arbitrary, it could be potentially dangerous for the drivers. Consequently, the degree of distortion must be regulated. As mentioned in the previous section, there are some standards that lateral-view mirror manufactures must follow. In particular, and in accordance with the JIS-D-5705 standard, a convex mirror must have a distortion factor lower than 5% with respect to its circular pattern that is projected on the mirror. The methodology defined by this standard to calculate this distortion factor is the following. A radial line pattern, as the one shown in Figure 2a, is placed at a distance of 300 mm in front of the mirror, as it is shown in Figure 2b. From this configuration, it is possible to acquire an image of the reflection of the radial pattern to analyze it and determine the distortion factor. According to the mirror distortion, the concentric circles will be deformed; i.e., the greater the distortion, the greater the deformation.

The way to calculate the distortion factor from the reflected images is performed by measuring eight distances from the points in the boundary of the *i*-th circle to the center; see Figure 2a. Then, the average of these distances must be calculated (${\bar{R}}_{i}$), and the maximum value must be obtained ($R_{0}$) to compute the absolute difference between ${\bar{R}}_{i}$ and $R_{0}$. Finally, the ratio between the aforementioned absolute difference and ${\bar{R}}_{i}$ is calculated. Equation (1) summarizes this procedure,

(1) $$\epsilon_{i}=\frac{|R_{0}-{\bar{R}}_{i}|}{{\bar{R}}_{i}}*100,$$

where $\epsilon_{i}$ is the distortion factor (%) of the *i*-th circle. The average of the measurements of the reflected image of the *i*-th concentric circle is calculated according to Equation (2),

(2) $${\bar{R}}_{i}=\frac{\left|\right|a_{i}^{\prime }{\left|\right|}_{2}+\left|\right|b_{i}^{\prime }{\left|\right|}_{2}+\left|\right|c_{i}^{\prime }{\left|\right|}_{2}+\cdots +\left|\right|h_{i}^{\prime }{\left|\right|}_{2}}{N},$$

where ${\bar{R}}_{i}$ is the average distance, whereas $\left\{a_{i}^{\prime },b_{i}^{\prime },c_{i}^{\prime },\cdots ,h_{i}^{\prime }\right\}$ represent the set of points (reflected by the mirror) at which the *i*-th circle and the radial lines intersect, N=8 is the number of points, and ∥·∥ refers to the $L^{2}$-norm. Note that if the image does not present any deformation, then the calculated distances correspond to the radius of the *i*-th circle. This procedure is performed for each one of the circles within the pattern. Then, if $\max\{\epsilon_{0},\epsilon_{1},\cdots ,\epsilon_{n}\}$ is greater than 5%, the shape does not satisfy the JIS-D-5705 standard.

## 3. Mirrors Distortion Calculation Based on Image Processing

**Figure 3:**
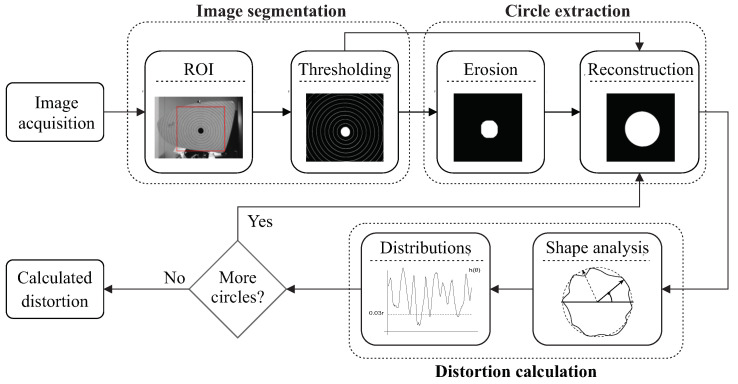
Block diagram of the proposed DCMIP.

In this work, a novel method to calculate the distortion in mirrors is presented. A general overview of the DCMIP is shown in Figure 3. As a first step, a gray-scale image of the radial pattern, following the setup established in the JIS-D-5705 standard (see Figure 2), is acquired. From this image, a region of interest (ROI) is selected, which will be used to identify all the concentric circles through a set of image processing algorithms, to then measure the distortion based on the circularity of each one of these circles.

All the steps of the block diagram are detailed in the following sections.

### 3.1. Image Acquisition

**Figure 4:**
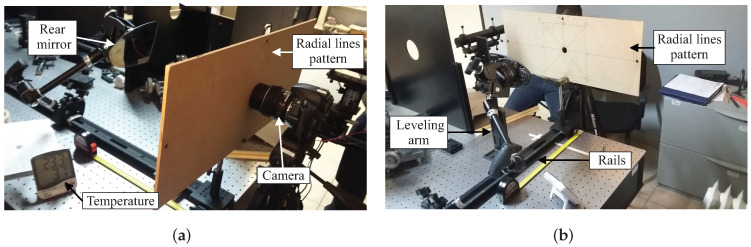
Experimental setup for image acquisition: (**a**) lateral view and (**b**) frontal view.

To guarantee that the image has not been distorted by the camera’s optics, a well-known camera calibration process has been performed to obtain both the intrinsic and extrinsic parameters of the camera [14]. This procedure is needed because the geometrical information of the mirrors will be computed. The experimental setup to acquire the image is shown in Figure 4. In the same way, to ensure the correct alignment between the camera and the circular pattern, a leveling arm holding the mirror and a mount holding the pattern were fixed by special rails on an optical table. The distance between both arms was at 300 mm, as set by the standard. Then, a laser pointer was used to verify that they are exactly aligned. If the mirror is not aligned with the camera, the circular patterns could present a misalignment and the experimental results would not be reliable.

### 3.2. Image Segmentation

**Figure 5:**
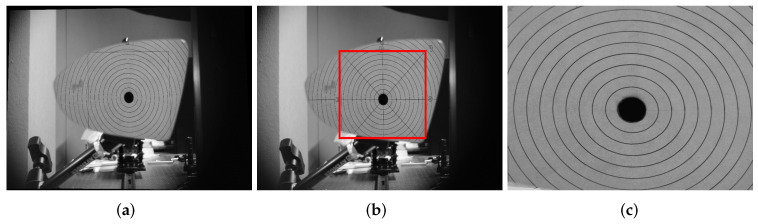
ROI selection and the resulting image. (**a**) Input image. (**b**) ROI selection. (**c**) Resulting image.

From a gray-scale image, a manually ROI selection is made. It is worth mentioning that this is the only user intervention during the whole process. In Figure 5, the selection of the ROI as well as the resulting image can be appreciated.

Now, let *I* denote the input image (for instance, the image shown in Figure 5c). Then, to separate the foreground (circles) from the background, a local thresholding approach is used; in this case, the Otsu method is the chosen one to perform this process [15]. In the local thresholding approach, the threshold value is determined by using the local information of the image to assign each pixel to the foreground or background.

**Figure 6:**
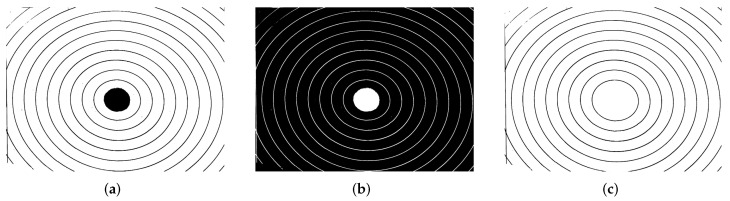
Segmented image. (**a**) Resulting image after the thresholding process. This image is denoted as $I_{b}$, (**b**) inverted binary image $I_{b^{\prime }}$, (**c**) this image is obtained after extract the first circle by applying next operator $I_{b}⋃J_{0}$.

Figure 6a shows the resulting segmented binary image ($I_{b}$), while Figure 6b shows the inverted binary image ($I_{b^{\prime }}$) of the previews one, which is need to perform the next step.

### 3.3. Circle Extraction

As mentioned in Section 1, a convex mirror will generate a distortion in the image it reflects. One way to determine how much distortion exists is by analyzing the reflected pattern. In this sense, in this work, a pattern of concentric circles has been used; therefore, it is essential to adequately extract these circles so that they can be used to determine the distortion. Then, to achieve the correct extraction, a morphological image analysis is used. The main morphological operations are dilation ($\delta_{\lambda B}$) and erosion ($\varepsilon_{\lambda B}$) where *B* represents the elementary structuring element in 2D containing its origin, and λ is a homothetic parameter. Therefore, the reconstruction of *X* from *Y* is obtained by iterating the elementary geodesic dilation of Y inside *X* until stability. In other words,

(3) $$R_{X}\left(Y\right)=\underset{n\geq 1}{⋃}\delta_{X}^{n}\left(Y\right).$$

**Figure 7:**
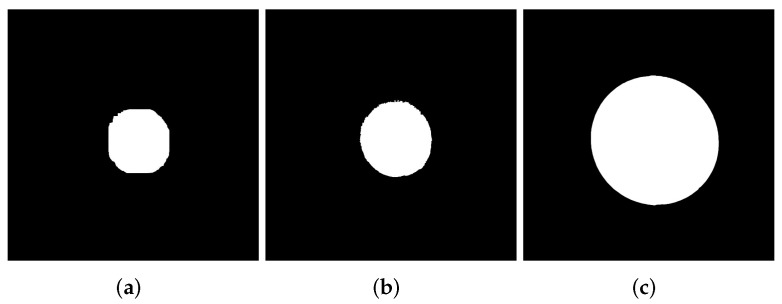
Circle extraction. (**a**) First circle partially extracted after erosion operation. (**b**) First circle completely reconstructed. (**c**) Second circle extracted.

Next, we present the proposed algorithm to segment the circles:

- $X=I_{b^{\prime }}$ denotes the input image. An example is presented in Figure 6b.
- Marker detection $Y=\varepsilon_{\lambda =10}\left(X\right)$. Figure 7a illustrates the eroded image.
- The reconstruction transformations $J_{0}=R_{X}\left(Y\right)$ is computed. The output image can be observed in Figure 7b.
- Measures are obtained from the first segmented circle.
- To extract the remaining circles, Equation (3) is applied successively. Now, the input image is $X=I_{b}⋃J_{0}$ (Figure 6c), and the markers to obtain the following circles are $Y=J_{i}$, with $J_{i}=R_{X}\left(J_{i-1}\right)$ for i>0. The image in Figure 6a shows the second circle. Measures are obtained from each segmented circle.

The segmented circles do not present new maxima or minima, since the reconstruction transformations are connected transformations.

### 3.4. Proposed Distortion Calculation

The proposed method is based on some of the ideas presented in the work of Herrera-Navarro et al. [8,13], in which a set of distances between the boundary of the shape and its centroid is used to generate a probability density function. In this work, instead of using this distance, a set of points that relates the boundary of the shape and the circle that best fits this shape is used. Such a shape can be described by the function $f:R\to R^{2}$ given by,

(4) $$f\left(\theta \right)=r\left(\theta \right)\cos\left(\theta \right)\;e_{1}\;+\;r\left(\theta \right)\sin\left(\theta \right)\;e_{2},$$

where $e_{1}$ and $e_{2}$ are the standard unit vector of $R^{2}$.

**Figure 8:**
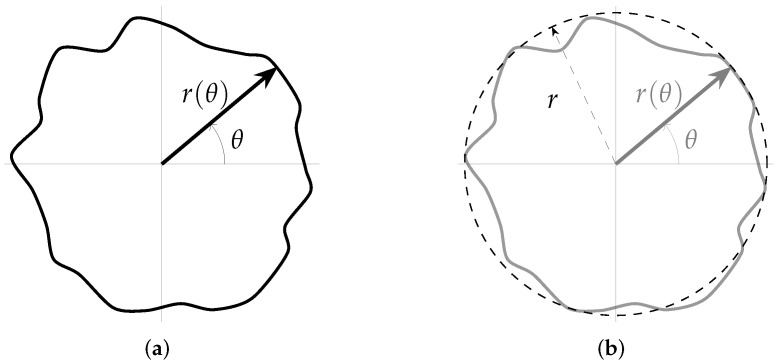
Example of a shape and its parameters. (**a**) Shape definition. (**b**) Best-fitted circle.

As Figure 8a depicts, the set of points of the shape is completely determined by θ and r(θ). Furthermore, $f_{n}\left(\theta \right)=∥f\left(\theta \right)∥$ can be considered as the “radius” distribution of the shape (where ∥·∥ is the vector norm).

**Figure 9:**
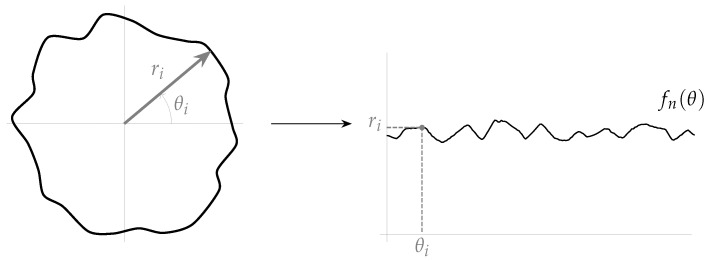
Radius distribution of the shape shown in Figure 8a.

Figure 9 shows the radius distribution ($f_{n}\left(\theta \right)$) of the shape shown in Figure 8a. From this distribution, the statistical mode (most frequently occurring value) is computed in such a way that the resulting value can be used as the radius (*r*) that best fits the shape. Nevertheless, considering that the distribution could be a multi-modal one, the mode with the larger value is used, and it is calculated as follows,

(5) $$r=\max_{m}\left\{m\in M:m=\mathrm{mode}(\;f_{n}\left(\theta \right)\;)\right\},$$

where *M* is the set that contains all the modes. In Figure 8b, the circle with radius *r* (calculating according to Equation (5)) that best fits the shape is shown. This circle is defined as follows,

(6) $$g\left(\theta \right)=r\cos\left(\theta \right)\;e_{1}\;+\;r\sin\left(\theta \right)\;e_{2}.$$

**Figure 10:**
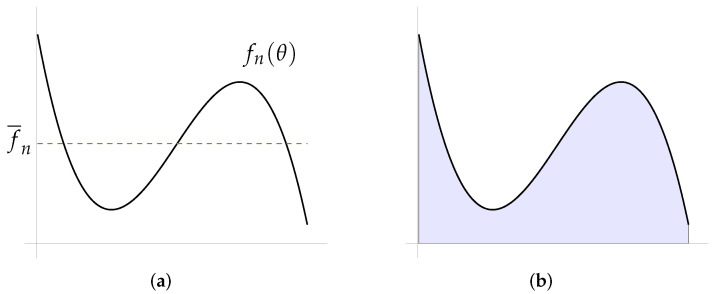
Case in which the ratio between the areas is an issue: (**a**) distribution given by $f_{n}\left(\theta \right)$ and (**b**) area under the curve.

A way of knowing how far the shape is from the ideal circle is through the calculation of the ratio between its distribution areas; however, this could be an issue. Suppose that the resulting $f_{n}\left(\theta \right)$ distribution is the one shown in Figure 10a, and the area under this curve is the one shown in blue in the Figure 10b. As can be seen, the area that is lost when the curve goes down is recovered when the curve goes up; that is, this area will be equivalent to the one computed below of the line positioned at the average value of the function (${\bar{f}}_{n}$). Now, suppose that the best-fitted circle distribution (∥g(θ)∥) is positioned at this average function value; then, the ratio of these two areas will tend to one, i.e., $Area\left(f_{n}\left(\theta \right)\right)/Area\left(∥g\left(\theta \right)∥\right)\to 1⟺r\to {\bar{f}}_{n}$, concluding that the shape “does not present” distortion with respect to the ideal circle.

**Figure 11:**
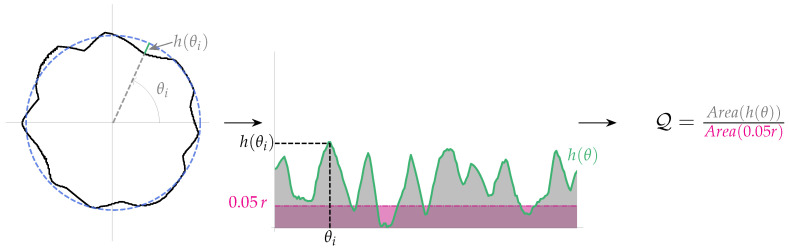
Distribution of the differences and the areas ratio. In “green”, the difference between shape and the ideal circle can be appreciated, while in “pink”, the difference between the ideal circle and the increased one is shown.

Consequently, in this work, the following solution is proposed. Given f(θ) and g(θ), the following function is defined,

(7) h(θ)=∥f(θ)−g(θ)∥

where ∥·∥ is the vector norm, while the function *h* can be interpreted as the distribution of the difference between the shape and the “ideal” circle. In addition, to include a certain tolerance threshold, the ideal circle is increased by 5% (this value was selected according to that established by the JIS-D-5705 standard). Then, the distance between the “ideal” circle (with radius *r*) and the increased circle (with radius 1.05r) is also calculated. Figure 11 shows both the h(θ) distribution as well as the distribution of the difference between the ideal circle and the increased one. As expected, the distribution between the difference of the ideal circle and the increased one must be constant.

Now, to determine if the shape is circular enough, the ratio of the area of h(θ) distribution and the area under the line given by 0.05r is calculated as follows,

(8) $$Q=\frac{\int_{a}^{b}\;h\left(\tau \right)\;d\tau }{\int_{a}^{b}\;0.05\;r\;d\tau }=\frac{1}{0.05\;\Delta \tau \;r}\int_{a}^{b}\;h\left(\tau \right)\;d\tau$$

where Δτ=(b−a). Thus, if Q≤1, then the shape is considered circular enough; i.e., its distortion is lower than the 5%, which is the value that the JIS-D-5705 standard considers as acceptable. This process is repeated for each of the circles that were extracted from the image; then, if for one of these circles, the ratio between areas (defined by Equation (8)) exceeds the value of one, then the mirror must be rejected.

## 4. Experimental Results and Discussion

To validate the proposed DCMIP, the calculation of the distortion factor of five commercial lateral-view mirrors (denoted as M1, M2, M3, M4, and M5) from different manufacturers was performed. It is worth mentioning that the mirrors are representative; that is, it is not intended to make a comparison between manufacturers but to have diversity to evaluate the performance of the proposed method. The experiments were performed following the experimental setup mentioned in Section 2. The images were taken with a Nikon D3300 camera with a resolution of 6000 × 4000 pixels (24MPix) and a Nikon AF-S Nikkor 18–55 mm F/3.5–5.6 lens, and the lighting and temperature conditions were controlled. The digital processing of the images and the calculation of the distortion were performed using Python.

The experimental results were divided into three sections. The first section shows the calculations of distortions of five lateral-view mirrors through the DCMIP. In the second section, a comparison between the DCMIP and the JIS-D-5705 standard is presented. Finally, the third section presents how the rotation of the shape affects the distortion measurement when using the standard, while the proposed method shows a constant behavior. Some other practical cases where the proposed DCMIP may be used are presented.

### 4.1. Results of the Proposed Distortion Calculation Method

**Figure 12:**
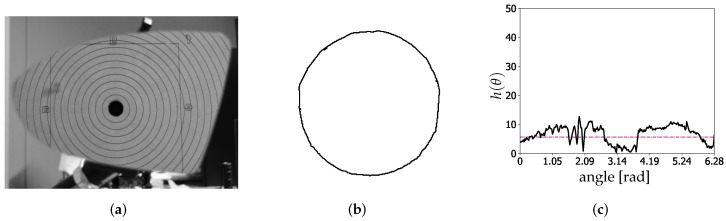
Representative calculation the distortion factor of M1. (**a**) Reflected circular pattern. (**b**) Extraction of the first circle. (**c**) Distribution of the difference between the extracted circle and the ideal one.

The performance of the proposed DCMIP to calculate the distortion of the mirrors is presented in this section. Figure 12a shows the image of mirror M2 captured by the camera with the reflected concentric circles pattern; Figure 12b depicts the extraction of one of the circles of the pattern, while Figure 12c illustrates the distribution of the difference between the extracted circle and the ideal one, i.e., h(θ). As explained in Section 3, from this distribution and the one generated by the difference between the ideal circle and the augmented one (by 5%), the respective areas under the curve are computed to then calculate the ratio between them.

**Figure 13:**
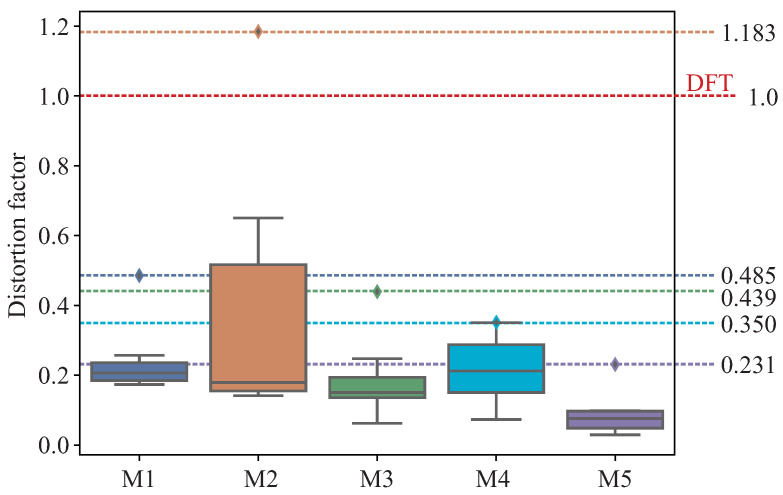
Distortion factor of 5 rearview mirrors using the DCMIP.

The box plot shown in Figure 13 summarizes the calculated distortion of the eight circles of the pattern for each mirror. As can be seen, the maximum distortion factor of M1, M3, M4, and M5 does not exceed the distortion factor threshold (DFT) equal to 1; that is, the distortion of the mirrors are less than 5%, as required by the JIS-D-5705 standard. On the contrary, the distortion factor of M2 exceeds the DFT; thus, the mirror has a distortion greater than 5%. Then, this mirror does not meet the standard.

### 4.2. Comparison between the JIS-D-5705 Standard and the DCMIP

For this comparison, the highest quality criteria were taken into account. On the one hand, as described in Section 2, the standard establishes that the maximum distortion of any reflected circle of the pattern must not exceed 5%. On the other hand, in the proposed DCMIP, the distortion factor of the pattern circles must not exceed the value of 1, which corresponds to the 5% established in the standard (see Section 3).

Table 1 shows the distortion of the five lateral-view mirrors under study. As it is shown, using the JIS-D-5705 standard, all the mirrors presented a distortion value lower than 5%; that is, their distortion is acceptable. On the contrary, through the proposed DCMIP, the mirror M2 presented a distortion factor greater than 1, which indicates that its distortion is greater than 5% and does not meet the minimum quality criteria. Mirrors M1, M3, M4, and M5 presented a maximum allowed distortion in both methods.

**Figure 14:**
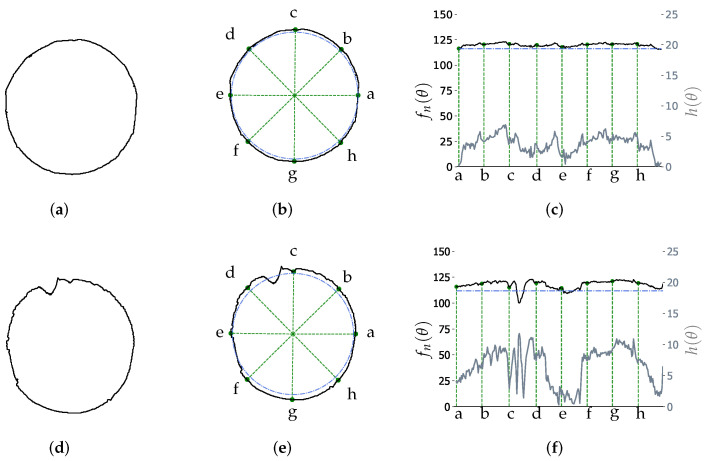
Examples of two circles of M2. (**a**) Shape of the first segmented circle. (**b**) Characteristic points to measure the distortion of the first circle, according to the standard. (**c**) Graphical representation of the distribution (first circle). (**d**) Shape of the second segmented circle. (**e**) Characteristic points to measure the distortion of the second circle, according to the standard. (**f**) Graphical representation of the distribution of distances (second circle).

To understand the differences in distortion measurements between both methods, an analysis of the distortion of M2 is presented. Figure 14a shows the shape of the first circle of the reflected pattern, which has slight variations from an ideal circle. According to the standard, a measurement of the radius of the “distorted circle” is taken every $45^{\circ }$ to later calculate the distortion by means of the procedure described in Section 2; as it is shown, with green dotted lines, in the Figure 14b. So, there are only eight measurements per circle; thus, the distortions that are not over the measurement points were not be considered. On the contrary, as illustrated in Figure 14c, the DCMIP uses a distribution of differences between the segmented circle and the ideal one to calculate a single distortion factor so that all distortions are taken into account. It can be seen that since the first circle does not present considerable distortions; then, the area under the curve described by h(θ) (gray line) is similar to the one defined by the difference of the augmented circle and the ideal one.

In the same way, the second circle of M2 was analyzed because it presented greater distortion, as can be seen in Figure 14d. Note that this circle has a large deformation between points c and d. As Table 1 shows, the M2 distortion using the standard was 2.798%. However, none of the points used to calculate this value passes over the greatest distortion of the circle (see Figure 14e); then, this deformation was not considered to calculate the standard measurement. This fact would allow accepting mirrors with small deformations and/or distortion that are not on the measurement points, which could put the safety of the driver at risk. In contrast, the proposed DCMIP did consider these zones, so the distortion factor was 1.183, and consequently, the M2 mirror did not pass the quality criteria. Figure 14f depicts, in a black line, the distribution of distances obtained as well as the distribution of differences between the distorted circle and the ideal one.

As mentioned in the previous analysis, in the JIS-D-5705 standard, the measurement points depend on the position of the shape; therefore, the distortion value will change if the shape rotates, while the distortion value obtained by DCMIP will not. The following section analyzes the rotation independence of the proposed DCMIP, which allows greater precision in the quality control of the mirrors.

### 4.3. Rotation Dependency Analysis

The rotation independence of the proposed DCMIP was validated using the second circle of M2. The results obtained were compared again with the JIS-D-5705 standard. The second circle of M2 has been chosen, since it presents some anomalies that were not initially considered by the standard. To analyze the performance, when a rotation has occurred, the distorted circle has been rotated from 0 to 45 degrees with variations of one degree.

**Figure 15:**
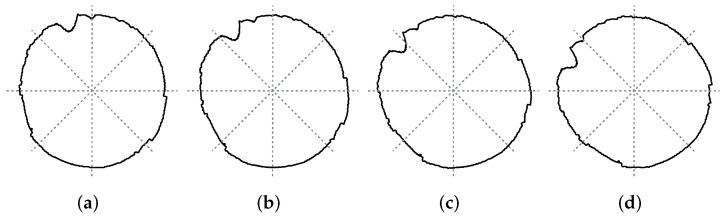
Rotation examples of the second circle of M2: (**a**) 0${}^{\circ }$, (**b**) 15${}^{\circ }$, (**c**) 30${}^{\circ }$, and (**d**) 45${}^{\circ }$.

Figure 15 shows four instances of these rotations, each one with 15 degrees of difference. This figure also shows how the measurement points, needed by the standard, change each time the circle is rotated; this means that the calculated distortion must also change.

**Figure 16:**
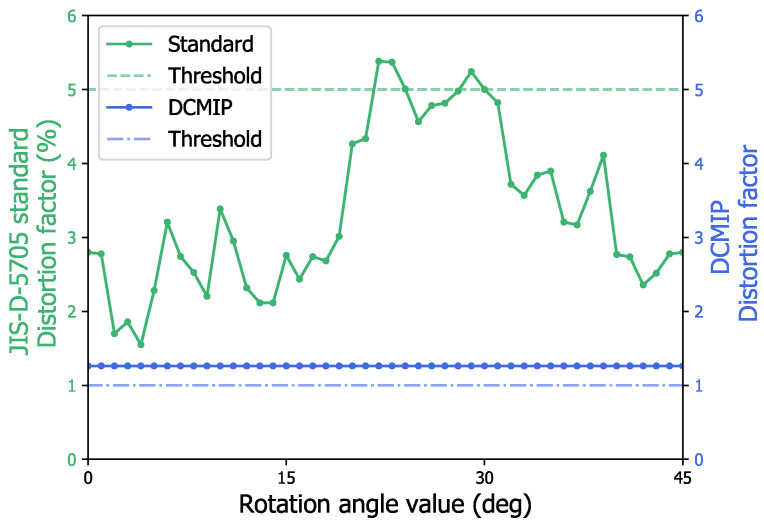
Comparative analysis of the deformation of the second circle of M2 by means of the JIS-D-5705 standard and the proposed DCMIP.

This behavior can be seen in the graph of Figure 16, in green color, which clearly shows how the value of the distortion factor changes according to the new orientation of the circle.

On the other hand, in Figure 16, it can also be appreciated, in blue, how the resulting distortion factor, calculated by the DCMIP, remains constant regardless of the orientation of the circle, indicating at all times that the mirror does not meet the minimum value of distortion allowed, while by using the standard method only when the circle has been rotated 23, 24, or 29 degrees, the distortion factor of the circle is greater than the minimum allowed value. Thus, this demonstrates that the JIS-D-5705 standard is not invariant to rotation, while the DCMIP is.

Considering the results obtained with the proposed method, it can be concluded that the DCMIP can be used in other applications where a measuring of the roundness and its deformation are required. For instance, in medicine as in [16,17], materials science [18,19], or even in digital image processing and discrete geometry [20,21], the proposed methodology can be used to measure circular objects and determine the deformation based on the desired criteria.

## 5. Conclusions

This work proposes a novel automatic method for calculating the distortion of an automobile lateral-view mirror based on image processing. The experimental results of the proposed DCMIP were compared with the JIS-D-5705 standard, which establishes that the maximum allowed side lateral-view mirror distortion is 5%, while the maximum allowed distortion factor of the DCMIP is equal to 1, which is equivalent to a distortion of 5%. Five commercial mirrors, from different distributors, were measured to validate the performance of the proposed method. Regarding the JIS-D-5705 standard, it was observed that the maximum distortion of the five mirrors was 3.166%; then, all mirrors meet the standard. In contrast, with the proposed DCMIP, the maximum distortion factor of one of the mirrors was 1.183 (which corresponds to the same mirror that presented the maximum distortion in the standard), indicating that the distortion is greater than 5% and does not meet the standard.

A detailed analysis of the effect that the rotation produces on the calculation of the distortion of the lateral-view mirror was carried out. It was observed that the procedure established in the JIS-D-5705 standard is highly susceptible to the rotation of the shape since the distortion is calculated with a set of eight angular equidistant points; therefore, some deformations may not be considered. This situation could affect the quality control of automobile lateral-view mirrors analyzed by the JIS-D-5705 standard, which could also put the safety of the driver at risk. On the contrary, the proposed DCMIP considers the contour of the concentric circle pattern so that the mirror distortions can be easily detected.

The results obtained showed that the DCMIP can measure distortions with high precision, and its effectiveness does not depend on the scale, resolution, or rotation of the image. In addition, unlike the standard, the calculation of the distortion does not depend on an expert person who performs the measurements and calculations manually.

## Figures and Tables

**Table 1:** Results of the distortion calculation of the mirrors using the JIS-D-5705 standard and the DCMIP.

Mirror	JIS-D-5705 Standard. Distortion Factor (%)	≤5%	Proposed DCMIP. Distortion Factor	≤1
M1	3.166	✓	0.485	✓
M2	2.798	✓	1.183	✗
M3	1.508	✓	0.439	✓
M4	1.889	✓	0.350	✓
M5	1.139	✓	0.231	✓
